# Attitudes, Beliefs, and Behaviors of Topical Antibiotic Prescribing among Primary Care Providers in Saudi Arabia: A Cross-Sectional Study

**DOI:** 10.3390/antibiotics13040301

**Published:** 2024-03-27

**Authors:** Baneen A. AlBeladi, Sara A. Alhubail, Riam A. Alsaqer, Ali N. Al-Nasser, Amira S. Radwan, Haytham A. Wali

**Affiliations:** Department of Pharmacy Practice, College of Clinical Pharmacy, King Faisal University, Al-Ahsa 31982, Saudi Arabiahwali@kfu.edu.sa (H.A.W.)

**Keywords:** topical antibiotics, primary care providers, antimicrobial resistance, antimicrobial stewardship, cross-sectional study

## Abstract

Background: The World Health Organization (WHO) estimates that 20–50% of antibiotics are misused in society. In addition to the development of antimicrobial resistance, topical antibiotics have been associated with adverse effects such as allergic contact dermatitis (ACD) and inadequate wound healing. This study investigated the appropriateness of topical antibiotic prescriptions among primary care providers in Saudi Arabia. Methods: A cross-sectional survey was conducted among Saudi Arabian primary care providers (physicians (general, family, and internal medicine)) employed in governmental and non-governmental healthcare facilities (primary care centers and outpatient clinics). Results: In total, 222 participants were included in the analysis. A total of 73% agreed that inappropriate topical antibiotic use puts patients at risk, and 43% reported antibiotic resistance in daily practice. Many respondents lacked knowledge of the proper indications for topical antibiotics, and 66.2% attributed this to a lack of updated knowledge, while 45% blamed inadequate supervision. Conclusion: Antibiotic prescription patterns deviated from the standards recommended by WHO. This calls for continuous review at all levels of healthcare, providing more physician education and ensuring that antibiotic therapy guidelines are easily accessible and effectively used to avoid the negative consequences of inappropriate antibiotic prescription.

## 1. Introduction

Prescribing medications is a challenging and essential practice that requires constant monitoring, evaluation, and adjustment. Additionally, it is based on the prescriber’s expertise, knowledge of medications, and comprehension of clinical pharmacology concepts. Antibiotics are drugs used to treat various local and systemic illnesses because they inhibit the growth of or destroy microbes. Antibiotics have significantly lowered morbidity and death rates due to infectious diseases. However, their indiscriminate use has increased antibiotic resistance and adverse pharmacological effects. The World Health Organization (WHO) estimates that 20–50% of antibiotics are misused in society [1]. The most prevalent reasons for the irrational use of medications include polypharmacy, incorrect use of antibiotics, excessive use of injectables, self-medication, and medication dispensing without adherence to clinical recommendations [2]. Because many organisms have developed resistance to widely used antibiotics, inappropriate antibiotic prescriptions pose a severe hazard to human health globally [3]. Methicillin-resistant *Staphylococcus aureus* (MRSA) is the subject of an alert worldwide, as its prevalence is higher than that of HIV/AIDS, emphysema, homicide, and Parkinson’s disease [4].

Healthy skin is a robust natural defense against disease invasion. Individuals with this compromised barrier may be more vulnerable to infections [5]. Physical trauma, such as abrasions, penetrations, cuts, burns, pre-existing dermatoses with compromised barrier functions, malnutrition, diabetes mellitus, and various congenital and acquired immunodeficiency disorders, can result in cutaneous bacterial infections [6].

The two most common organisms causing primary and secondary infections of the skin and minor skin wounds are *Staphylococcus aureus* and group A streptococci [7]. Gram-negative organisms, primarily enteric bacilli (*Pseudomonas aeruginosa*), are occasionally responsible for skin infections, foot ulcers, and cutaneous infections, particularly in the groin and ears. Skin infections are frequently prevented and treated with topical antibiotics [6].

Topical antibiotics are frequently used to treat or prevent infections after minor cuts, abrasions, burns, surgical wounds, and superficial pyoderma such as impetigo [6]. Topical antibiotics may have a minimal or moderate effect when treating folliculitis and furuncles to prevent the spread of the infection from the original lesion to nearby follicles [8]. The best way to treat other pyodermas, including carbuncles, ecthyma, cellulitis, or erysipelas, is with systemic antibiotics [6]. Secondary bacterial infections associated with skin diseases, including eczema and leg ulcers, are typically treated with topical antibiotics [6]. After minor surgery, topical antibiotics can be used to clean wounds and promote healing or prevent infections [9]. Burns frequently receive prophylactic topical treatment to avoid the complications of severe secondary infections [6].

In addition to the development of antimicrobial resistance (AMR) [10], topical antibiotics are associated with adverse effects such as allergic contact dermatitis (ACD) and inadequate wound healing [11]. Since no existing study has shed light on skin topical antibiotic appropriateness among primary care providers in Saudi Arabia, this study aimed to investigate the appropriateness of topical antibiotic prescription among primary care providers in Saudi Arabia.

## 2. Methods

### 2.1. Setting, Design, and Population Sampling

This cross-sectional study was conducted in Saudi Arabia between December 2022 and February 2023. Simple random sampling was used. The study targeted primary care providers (physicians (general, family, and internal medicine)) aged 25 years or older who were employed in governmental and non-governmental healthcare facilities (primary care centers and outpatient clinics in hospitals). Interns, secondary care providers, and tertiary care providers were excluded.

### 2.2. Sample Size

Using the online Raosoft^®^ sample size calculator (Raosoft Inc., Seattle, WA, USA) and based on a 95% confidence interval and a 5% margin of error with a total of 29,121 estimated primary healthcare providers in Saudi Arabia (according to the 2021 Ministry of Health (MOH) data), a minimum sample size of 378 participants was needed for this study. Therefore, the required sample size was rounded off to 400 participants.

### 2.3. Questionnaire

**Figure 1:**
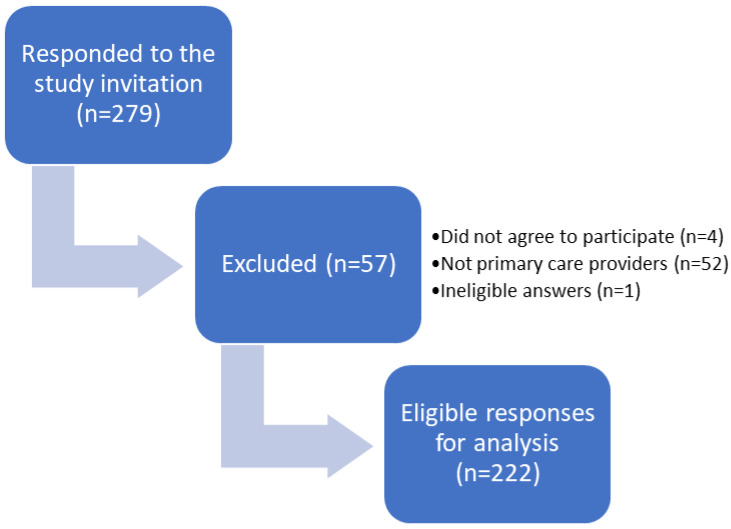
Flow Diagram of the Study Participation.

We constructed a new questionnaire adapted from an earlier questionnaire that assessed antibiotic appropriateness [12]. A panel of experts evaluated and validated the questionnaire. Eleven primary care providers assessed the readability and clarity of the questionnaire. Based on the pilot study, the questionnaire was modified and refined to the final version, which consisted of 34 questions that assessed five main domains (demographic data, knowledge and attitude toward topical antibiotic use, practices of antibiotic prescribing, accessibility and use of antibiotic therapy guidelines, and appropriateness of topical antibiotic prescription for the most common indications. We published an online Google Forms questionnaire from December 2022 to February 2023. In addition, the authors visited several primary clinics and distributed the questionnaires to family medicine physicians. Ineligible participants were excluded, as shown in the study participation diagram (Figure 1).

### 2.4. Statistical Analysis

Descriptive statistics were used to summarize the findings of the study. Categorical variables were presented as frequencies and percentages. Parametric continuous variables were presented as means and standard deviations (SD), while non-parametric continuous variables were presented as medians and interquartile ranges (IQR). Cronbach’s alpha test was conducted to assess the reliability of each section’s answers. Statistical analyses were performed using IBM SPSS Statistics (version 27.0, IBM Corp., Armonk, NY, USA).

## 3. Results

Table 1 summarizes the sociodemographic characteristics of the study participants. The responses of 222 participants (89 males and 130 females) were included in the analyses (Figure 1). The mean age of the participants was 36.8 years (±10.31) and they were from different places of residence in Saudi Arabia. Approximately half of the healthcare providers were family medicine physicians (107, 48%) and their professional titles were general practitioners (79, 36%), residents (67, 30%), specialists (60, 27%), or consultants (16, 7%). Ministry of Health hospitals were the most common primary care sites (160, 68%), followed by university hospitals (34, 14%). Local antibiograms were not available in more than half of the primary care physicians’ worksites (127, 57%); however, slightly more than half of the participants (126, 57%) had local antibiotic prescription guidelines, in which the majority (105, 83%) were easily accessible.

Most primary healthcare providers (163, 73%) agreed that prescribing topical antibiotics inappropriately puts patients at risk and the same number disagreed that everyone should be able to buy topical antibiotics without a prescription (Table 2). About two-thirds of primary care providers (155, 70%) agreed that over-prescription of topical antibiotics is not always better than under-prescription. Opinions regarding antibiotic resistance were divided, as 96 and 79 participants reported that antibiotic resistance was an issue in their daily practice or healthcare facility, respectively. In comparison, 84 and 93 participants found that antibiotic resistance was not an issue in their daily practice or healthcare facilities, respectively.

Approximately one-third of participants (66.2%) indicated that their patient’s clinical condition was the most crucial factor influencing their decision to initiate topical antibiotic therapy. However, positive microbiological results in symptomatic patients were vital for fewer than half of the respondents (46.4%). More than half (55%) of the participants reported trying to keep their topical antibiotic prescription cost-effective. Regarding the reasons for the inappropriate use of topical antibiotics, the participants believed that un-updated knowledge (66.2%) and inadequate supervision (45%) were the main factors that significantly contributed to the inappropriate use of topical antibiotics. The most frequently mentioned solutions for topical antibiotic resistance were physician education on appropriate antibiotic therapy (65.8%) and the provision of local antibiotic guidelines (57.6%). It should be mentioned that less than half of the participants (47.7%) had regular training and education on antibiotic prescription in their workplace. (Table 3).

Approximately one-third of participants chose incorrect indications for metronidazole (34.5%), clindamycin (34%), or silver sulfadiazine cream (32%) (Table 4). Most primary care providers participating in the study (84.4%) could not recognize the proper indications for neomycin (Betnovate-N, 0.1%). Similar results were observed for fusidic acid plus betamethasone (Fucicort) (85%) and fusidic acid plus hydrocortisone (Fucidin H) (78%). However, most participants (71.7%) correctly identified the indications for fusidic acid alone (Fucidin). Conversely, almost half of the participants (43%) were able to identify the use of mupirocin (Avoban cream) correctly, and a greater proportion correctly identified benzoyl peroxide (Benzac AC gel) (56%) and neomycin and bacitracin (Baneocin cream) (57%).

## 4. Discussion

This study evaluated the knowledge, attitudes, and prescription habits of topical antibiotics among Saudi Arabian healthcare professionals. No prior research has concentrated on the prescription habits of healthcare providers of topical antibiotics. Healthcare professionals are vital players in the misuse of antibiotics and the emergence of antibiotic resistance. There is a need for them to better understand proper topical antibiotic prescription practices [13].

In the present study, there was a scarcity of available local antibiograms. The MRSA prevalence among the facilities with antibiogram data (8%) was greater than 20% at their sites, and 38% of the facilities did not have antibiogram data available at their sites. In one-third of the hospitals (29%), local antibiotic prescription guidelines were unavailable; in the hospitals where the guidelines were available, 17% stated that accessing these clinical guidelines was challenging. This study showed a connection between a lack of knowledge and incorrect prescription practices. This could explain why some healthcare providers never followed the antibiotic guidelines, never providing them with safe and effective clinical recommendations. Instead, they persist in inappropriately prescribing antibiotics, which could be a factor in the rising rates of antibiotic resistance.

We compared our study to previous studies based on resistance and antibiogram availability and found one study on the resistance rates of *Cutibacterium acnes* (formerly *Propionibacterium acnes*). Approximately half of the patients with *C. acne* became resistant to oral or topical treatments, and one in four *C. acne* strains was resistant to tetracyclines, macrolides, or clindamycin. Nevertheless, recent studies showing rising levels of resistance suggest that this could change in the future [14]. Another study investigated the susceptibility of MRSA, *Acinetobacter baumanii-calcoaceticus* (ABC), extended-spectrum beta-lactamase (ESBL)-producing *Klebsiella pneumoniae*, and *Pseudomonas aeruginosa* to topical antimicrobial agents. However, unlike other studies, they did not find that multidrug-resistant (MDR) isolates were more resistant to topical treatments than non-MDR isolates [15].

A previous study revealed that local antibiotic prescription guidelines were scarce and difficult to access [12]. Our research yielded different results, with more than half (57%) of the participants indicating that the guidelines were available and the majority (83%) stating they were easily accessible. The difference between the results of the two studies may be attributed to the disparity in healthcare quality between the regions in which they were conducted.

A previous study revealed that more than half of healthcare providers (51.2%) strongly agreed that inappropriate prescription of antibiotics puts patients in danger [12]. This aligns with our study results, as more than two-thirds of the healthcare providers (73%) acknowledged that incorrect prescription of topical antibiotics can be dangerous for patients, demonstrating an understanding of the potential harm associated with their use. Nevertheless, only a small proportion of healthcare providers (19%) disagreed with this statement, indicating a possible lack of awareness or knowledge regarding the dangers of misusing topical antibiotics. This underscores the importance of ongoing education and training of healthcare professionals to ensure proper antibiotic prescription. Additionally, the study revealed that most healthcare providers (73%) disagreed that topical antibiotics should be sold without prescription. This outcome implies that healthcare professionals in Saudi Arabia are aware of the need to control the use of topical antibiotics to avert their misuse and potential damage.

Furthermore, the study revealed that most healthcare providers (70%) disagreed with the notion that over-prescribing topical antibiotics is always preferable to under-prescribing them. This finding suggests that healthcare professionals in Saudi Arabia are aware of the potential risks of overusing antibiotics and prioritize patient safety over the possible advantages of over-prescribing them. Ultimately, the study revealed that more than one-third of healthcare providers (36%) concurred that antibiotic resistance to topical treatments is a significant problem in healthcare facilities. This finding underscores the importance of ongoing surveillance and monitoring of antibiotic resistance in Saudi Arabia to guide appropriate prescription practices and impede the spread of antibiotic-resistant infections. These discoveries emphasize the need to create and execute antimicrobial stewardship programs to encourage using suitable antibiotics in Saudi Arabia.

More than two-thirds of the participants (66.2%) stated that un-updated knowledge was a significant factor in the inappropriate use of topical antibiotics. However, less than half of the respondents (45%) believed inadequate supervision contributed to improperly using topical antibiotics. It is crucial to highlight that we did not attempt to evaluate the appropriateness of the prescription; instead, we concentrated on physicians’ opinions and perceptions to assist in planning the correct prescription of topical antibiotics. A previous study reported that 29% of participants identified a lack of interest in antibiotic prescriptions and infection management as the cause of inappropriate antibiotic use [12]. Our study found that a similar percentage (30.6%) of participants reported that a lack of interest in antibiotic prescriptions and infection management was not an essential factor in the inappropriate use of topical antibiotics. Our findings underline the need for well-thought-out educational and training programs to fill the knowledge gaps and promote proper evidence-based prescription practices for topical antibiotics. For more than half of the participants (55%), cost-effectiveness was mainly considered when making topical antibiotic prescription decisions, whereas less than half (31%) did not consider cost-effectiveness when prescribing. Most respondents (65.8%) believed physician education on proper antibiotic therapy might help reduce topical antibiotic resistance. More than half of the respondents (57.6%) reported that providing local antibiotic guidelines was another factor that may help mitigate topical antibiotic resistance. Furthermore, 52.3% of participants reported not receiving regular training or education in their workplaces. Compared with a previous study conducted in Tanzania, 24% of the respondents did not receive regular training [12]. Therefore, it is important to consider training and education programs regarding appropriate topical antibiotic prescriptions to minimize antibiotic resistance.

Among the participants, 65.4%, 66%, and 68% reported inappropriate use of metronidazole, clindamycin, and silver sulfadiazine, respectively. The results indicate that a large number of healthcare providers prescribe metronidazole for other skin disorders rather than for the appropriate indications (trichomoniasis and rosacea, acne vulgaris, and burns (second- or third-degree)). This highlights that a large number of patients were under-treated. This is consistent with a study that assessed community pharmacists’ antimicrobial resistance awareness, antibiotic prescription errors, and dispensing patterns, and highlighted that 28.7% of general practitioners inappropriately prescribed antibiotics and 4.6% of internal medicine specialists [14]. A total of 84.4% of neomycin (Betnovate-N 0.1% cream) responses were inappropriate, indicating that the drug was misused because of a lack of knowledge. Appropriate indications for mupirocin (Avoban cream) (infected wounds (abrasion) and impetigo) were chosen by 43.4% of the respondents. Therefore, there is a compelling need for awareness regarding mupirocin indications.

The most inappropriately used topical antibiotic was fusidic acid plus betamethasone (Fucicort). The result demonstrates that the Fucicort-only approved indication (corticosteroid-responsive dermatoses with secondary infection) was only known by 15% of the respondents. Fusidic acid 5% (Fucidin cream) was appropriately used by 71.7% of the participants. Impetigo, erythrasma, infected wounds (abrasion), and infected burns appear to be appropriately managed with Fucidin cream. Regarding Fucidin H, it was the second most inappropriately used antibiotic, as 78% of the respondents chose inappropriate indications. The risk of increased MRSA resistance to fusidic acid is expected to be high [10] and the reported improper use of this commonly used topical antibiotic is alarming. It is worth noting that misdiagnosis of skin infections is widespread [16,17]. Many primary care doctors over-diagnosed skin infections and under-diagnosed inflammatory conditions [18,19]. Fusidic acid corticosteroid combinations are not superior to the corticosteroid in infected or noninfected atopic dermatitis [20].

This study had certain limitations. First, we used a target sample size of 400 participants. Nevertheless, we stopped collecting data once the questionnaire reached saturation, with a final response count of 222. In addition, Cronbach’s alpha test results were satisfactory. This suggests that the questionnaire needed to be more reliable. All 34 questions in the questionnaire had 222 responses, except for the gender question, which had 219. This was because the question had been altered to an optional one. Second, we could not identify any correlation between specific knowledge areas and certain behaviors, which could have been useful in pinpointing the source of the problem. Third, our study did not specifically investigate the effect of inappropriate use of topical antibiotics and antimicrobial resistance, and the literature on this topic revealed conflicting results. Further studies are needed to address this issue. Finally, the questionnaire method implies that physicians provide their opinions only. Some physicians are unable to justify the suitability of topical antibiotics accurately. They may require guidance or be introspective to confirm the inappropriateness. Consequently, the study findings and conclusions ought to be carefully interpreted.

## 5. Conclusions

The antibiotic prescription pattern in this study did not adhere to the standards that the World Health Organization (WHO) has recommended. To prevent the adverse effects of incorrect antibiotic prescriptions, ongoing assessment at all levels of healthcare, increased physician education, and ensuring that antibiotic therapy guidelines are readily available and adequately implemented are necessary.

## Figures and Tables

**Table 1:** Baseline Characteristics of the Study’s Participants (*n* = 222).

Value	Variable
	Gender, *n* (%)
89 (41)	Male
130 (59)	Female
36.8 (10.31)	Age, mean (SD)
	Geographical region, *n* (%)
74 (33)	Eastern region
49 (22)	Western region
17 (8)	Central region
37 (17)	Northern region
45 (20)	Southern region
	Specialty, *n* (%)
84 (38)	Non-specialist
107 (48)	Family medicine
31 (14)	Internal medicine
	Professional title, *n* (%)
79 (36)	General practitioner
67 (30)	Resident
60 (27)	Specialist
16 (7)	Consultant
	Primary care site, *n* (%)
160 (68)	Ministry of Health hospitals
14 (6)	National Guard hospitals
17 (7)	Ministry of Defense hospitals
3 (1)	Security Forces hospitals
34 (14)	University hospitals
5 (2)	King Faisal Specialist Hospital and Research Center
4 (2)	Private hospital
	Years of experience, *n* (%)
87 (39)	0–4 years
52 (23)	5–9 years
33 (15)	10–14 years
24 (11)	15–19 years
26 (12)	>20 years
	Availability of a local antibiogram, *n* (%)
53 (24)	Yes
127 (57)	No
42 (19)	I don’t know
	MRSA prevalence among antibiogram-available facilities, *n* (%)
13 (25)	Less than 10%
16 (30)	Between 10 and 20%
4 (8)	More than 20%
20 (38)	Unavailable in my site
	Availability of local antibiotic prescribing guidelines, *n* (%)
126 (57)	Yes
65 (29)	No
31 (14)	I don’t know
	Accessibility of guidelines, *n* (%)
105 (83)	Easily accessible
21 (17)	Difficult

MRSA: methicillin-resistant *Staphylococcus aureus;* SD: standard deviation.

**Table 2:** Knowledge of Primary Care Providers of Topical Antibiotics Prescribing.

Parameter	Value, *n* (%)		
	Yes	No	I Don’t Know
Does inappropriate prescribing of topical antibiotics put patients at risk?	163 (73)	43 (19)	16 (7)
Is it always better to over-prescribe topical antibiotics than to under-prescribe?	43 (19)	155 (70)	24 (11)
Should everyone be able to buy topical antibiotics without a prescription?	32 (14)	163 (73)	27 (12)
Is topical antibiotic resistance an issue in your daily practice?	96 (43)	84 (38)	42 (19)
Is topical antibiotic resistance a significant issue in your healthcare facility?	79 (36)	93 (42)	50 (23)

**Table 3:** Practices of Primary Healthcare Providers in Prescribing Topical Antibiotics (*n* = 222).

Parameter	*n* (%)
Which of these factors may influence your decision to start topical antibiotic therapy? *	
Patient’s clinical condition	147 (66.2)
Patient’s preference	44 (19.8)
I was trained to prescribe topical antibiotics	44 (19.8)
Positive microbiological results in symptomatic patients	103 (46.4)
Wanting to satisfy the senior treating physician	21 (9.5)
Worry of missing patients with possible infections	46 (20.7)
Do you ever try to ensure that your topical antibiotic prescribing is cost-effective?	
Yes	123 (55)
No	69 (31)
I don’t know	30 (14)
Which of these do you think are important causes of prescribers’ inappropriate use of topical antibiotics? *	
Un-updated knowledge	147 (66.2)
Unrestricted availability of antibiotics	72 (32.4)
Inadequate supervision	100 (45)
Lack of interest in the subject of antibiotic prescribing and infection management	68 (30.6)
Strained healthcare personnel	44 (19.8)
Which of the following do you think may help minimize topical antibiotic resistance? *	
Treating infection, not contamination or colonization	82 (36.9)
Physician education on appropriate antibiotic therapy	146 (65.8)
Consulting with infectious diseases experts	88 (39.6)
Providing local antibiotic guidelines	128 (57.6)
Knowledge of pathogens and antibiotic susceptibility test results	112 (50.5)
Restrict topical antibiotic use	61 (27.5)
Have you received regular training and education in antibiotic prescribing in your workplace?	
Yes	106 (47.7)
No	116 (52.3)
Do you follow the recommendations of your healthcare facility guidelines on topical antibiotic prescribing?	
Yes	119 (94)
No	7 (6)

* This question was formulated as a multiple-choice multiple response (MCMR) question. Therefore, each choice item of the question was calculated as a stand-alone response out of the total of 222 responses (i.e., adding the response percentages of all the items will add up to more than 100).

**Table 4:** Appropriateness of Topical Antibiotic Prescribing for the Most Common Skin Diagnoses.

Indications		Topical Antibiotic (Brand Name)
Inappropriate	Appropriate	
N (%)	N (%)	
225 (65.4)	119 (34.5)	Metronidazole (Rozex 0.75% cream)
220 (66)	114 (34)	Clindamycin (Avocin cream)
215 (68)	103 (32)	Silver sulfadiazine (Flamazine cream 1%)
282 (84.4)	52 (15.5)	Neomycin (Betnovate-N 0.1% cream)
180 (56.6)	138 (43.4)	Mupirocin (Avoban cream)
331 (85)	58 (15)	Fusidic acid and betamethasone (Fucicort)
118 (44)	151 (56)	Benzoyl peroxide (Benzac AC gel)
117 (28.3)	297 (71.7)	Fusidic acid 5% (Fucidin cream or ointment)
283 (78)	81 (22)	Fusidic acid and hydrocortisone (Fucidin H)
152 (42.8)	203 (57.1)	Neomycin and bacitracin (Baneocin)

## Data Availability

The data presented in this study are available on request from the corresponding author.
